# Citicoline – a neuroprotector with proven effects on glaucomatous disease

**DOI:** 10.22336/rjo.2017.29

**Published:** 2017

**Authors:** Chitu Iulia, Tudosescu Ruxandra, Leasu-Branet Costin, Voinea Liliana-Mary

**Affiliations:** * Department of Ophthalmology, University Emergency Hospital, Bucharest, Romania

**Keywords:** Citicoline, neuroprotection, glaucoma

## Abstract

Citicoline is the generic name of cytidine-5’-diphosphocholine (CDP-choline), an endogenous compound that is able to increase the levels of neurotransmitters in the central nervous system by interacting with the synthesis of cellular membranes phospholipids, especially phosphatidylcholine. Exogenous Citicoline, administered by ingestion or injection, is hydrolyzed and dephosphorylated in order to form cytidine and choline, which resynthesize CDP-choline inside brain cells. It has proven neuroprotective effects in Alzheimer disease, stroke, and Parkinson’s disease, as well as in glaucoma and amblyopia. Citicoline acts as a neuroprotector for those patients with progressive glaucomatous disease in spite of well-controlled intraocular pressure. The purpose of this review was to outline the main features of Citicoline and the evidences of its effect in glaucoma.

## Pharmacology and effects

Cytidine-5’-diphosphocholine (CDP-choline, CDPCho) or Citicoline is a pharmaceutical substance identical with the natural compound, and has an important role in the phospholipid synthesis. In 1050s, Kennedy and collaborators showed that Citicoline is a precursor of phosphatidylcholine (PC), one of the most important phospholipid of the cell membrane [**1**].

Phospholipids are major constituents of cell membrane, with a high turnover rate, thus the continuous synthesis of these substances ensure the optimal structure and function of the cell.

**Fig. 1:**
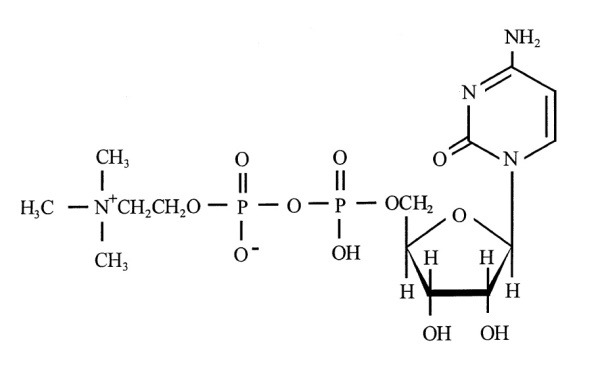
Chemical structure of Citicoline

CDP-Choline is a mononucleotide made of ribose, pyrophosphate, cytosine and choline, and its chemical structure is illustrated in **Fig. 1** [**2**].

**Fig. 2:**
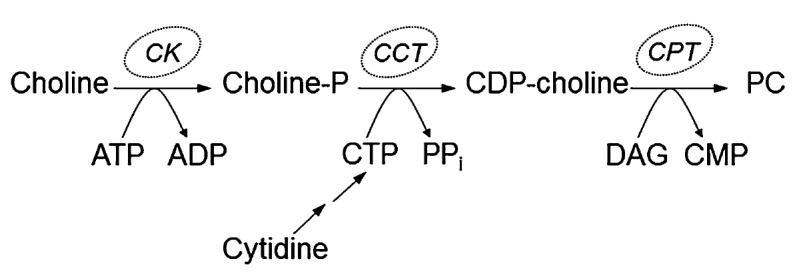
The CDP-choline pathway

The CDP-choline pathway is the pathway of the novo synthesis of phosphatidylcholine and includes the enzymes cytidine kinase (CK), choline phosphate cytidilyltransferase (CCT) and CDP-choline:1,2-diacylglicerol choline phosphotransferase (CTP). In this pathway, choline is provided by PC turnover and transport into the cell (**Fig. 2**) [**1**].

The term “Citicoline” was first introduced in the 1970s when the substance was used as a drug. Manaka et al. from Japan administered Citicoline for the first time in 1974 for Parkinson’s disease.

When administered (orally or parenterally), Citicoline is quickly metabolized (minutes) and transformed in pyrimidinergic and cholinergic catabolites. It is a safe molecule, with only few adverse effects, such as digestive intolerance after oral administration. The therapeutic dosage in humans is 500-2000 mg Citicoline daily (7-28 mg/ kg) [**1**].

The most important phospholipids are phosphatidylinositol, phosphatidylethanolamine, phosphatidylcholine, and sphingomyelin. They are part of cell membrane and ensure its function, like enzymatic processes of membrane, linking receptors and intracellular signals, and maintaining the cell homeostasis. The mechanism of some neurodegenerative diseases, such as vascular dementia, Alzheimer disease, and cognitive impairment involve specific changes in neuronal membrane and metabolism of structural phospholipids. The apoptotic cascade is triggered by phosphatidylcholine metabolism changes. CDP-choline is also linked to acetylcholine metabolism. Thus, exogenous Citicoline administration provides choline for acetylcholine synthesis [**2**].

**Pharmacokinetics**

When administered orally or intravenous, Citicoline is converted to cytidine and choline, its main circulating metabolites. Plasmatic cytidine is then converted to uridine, and, this results in uridine phosphate inside the brain. At neuronal level, this is transformed to cytidine triphosphate. After administration, Citicoline rapidly diffuses to the tissues and is actively used. It can be found in liver, brain, and kidney. The excretion of CDP-choline is by urinary or fecal route and in expired CO2.

Citicoline provides neuroprotection by few mechanisms such as the following: maintains sphingomyelin and cardiolipin levels (constituent of inner mitochondrial membrane), restores the phosphatidylcholine (PtdCho) levels, increases the activity of glutathione reductase activity and the glutathione synthesis, decreases lipid peroxidation, and restores the activity of Na+/K+ ATPase. It is also involved in acetylcholine synthesis, providing choline [**3**].

Cytidine and choline are the two parts of Citicoline connected by a diphosphate bridge. After being absorbed, cytidine and choline are re-phosphorylated, and Citicoline is recreated from choline monophosphate and cytidine triphosphate.

In the course of PtdCho synthesis, choline monophosphate is added to PtdCho, releasing cytidine 5’-monophosphate (CMP). CMP can be used for DNA or RNA synthesis. The acetylation of choline part of Citicoline leads to acetylcholine.

Studies have shown that Citicoline is almost completely absorbed when it is orally administered and only a small part of it is excreted. Citicoline metabolites reach the brain in about 30 minutes after administration, but the blood level has a slow increase, with a peak at 6 hours after the oral intake.

The choline is used by cholinergic neurons in two metabolic pathways: synthesis of PtdCho and the neurotransmitter acetylcholine. The two pathways compete for the available choline, which is used preferentially for acetylation. When choline is depleted, phospholipids (PtdCho) are hydrolyzed in order to restore choline levels. Acetylcholine synthesis is favored when the available amount of choline is limited. Therefore, Citicoline is a source of choline, avoiding PtdCho hydrolysis and cholinergic neurons death.

Choline liberated from Citicoline can be metabolized to glutathione, one of the most important endogenous antioxidant defense systems in the brain. Glutathione has a neuroprotective role by decreasing lipid peroxidation. Citicoline has proven effects in cerebral edema reduction by restoring Na+/K+-ATPase activity in traumatic brain injury and transient focal or global ischemia [**3**].

Having these properties, Citicoline has been studied as a promising therapeutic agent in brain ischemia, Parkinson’s disease, Alzheimer’s disease and ocular diseases such as glaucoma, non-arteritic ischemic neuropathy and amblyopia.

**Use of Citicoline in neurological diseases – evidence**

Many experimental studies have proven the protective effects of Citicoline in stroke models by reducing the infarct volume and brain edema, leading to improvement of neurological deficits. In these studies, Citicoline was used as a single therapy or in combination with others agents [**4**]. Moreover, the benefic role of Citicoline was reported in clinical stroke trials when administered soon after ischemia.

In Alzheimer’s disease, Citicoline may inhibit the deposition of beta-amyloid, a neurotoxic protein involved in the pathophysiology of the disease [**5**]. It is believed to be an interaction between the formation of amyloid peptides and membrane phospholipids disintegration.

Alvarez et al. [**6**] revealed in a study performed on 30 patients with Alzheimer’s disease that after 12 weeks of treatment with Citicoline, the cognitive performance increased, and this was more pronounced in patients with mild dementia. It was also an increase of cerebral blood flow velocity and of brain bioelectrical activity.

The improvement in mental performance and brain electrical activity was also proven by Franco et al. [**7**] after one month of treatment with Citicoline in patients with an early onset of Alzheimer’s disease.

Many authors have studied the role of Citicoline in Parkinson’s disease [**8**-**10**] and concluded that the basis of symptoms improvement is the stimulation of the dopaminergic system.

Agnoli et al. [**8**] administered Citicoline to patients with Parkinson disease already treated with L-dopa + dopa decarboxylase inhibitor and revealed that Citicoline determined an important improvement of bradykinesia and rigidity. Citicoline could decrease the incidence of side effects and slow down the loss of efficacy of levodopa in the long-term treatment [**9**].

**Use of Citicoline in Amblyopia and Non-arteritic Ischemic Optic Neuropathy**

In amblyopia, Citicoline may improve the retinal and postretinal visual pathways by stimulating the dopaminergic system. It has been proven that it enhances contrast sensitivity, visual acuity, visual evoked responses and the effect of part-time occlusion.

Campos et al. [**11**] treated children with amblyopia (anisometropic or strabismic amblyopia) with oral Citicoline in addition to patching. After 30 days of treatment, the conclusion was that Citicoline was not more effective than patching alone but was able to stabilize the effect obtained during the treatment. They maintained the same visual acuity after 90 days compared to those who had only patching and who showed a decrease of visual acuity [**12**,**13**].

Another study made by Porciatti et al. [**14**] also showed the benefic role of Citicoline in amblyopia. He treaded 10 amblyopic patients with intramuscular Citicoline for 15 days. The visual acuity improved in both eyes with 1.4-1.5 lines and with 0.4 lines in the control group. The contrast sensitivity and the visual evoked potential also improved.

In 2008, Parisi et al. [**15**] proved the positive role of Citicoline treatment on patients with non-arteritic ischemic optic neuropathy. He evaluated the visual function before and after a 60 days treatment with oral Citicoline. There was an improvement of visual acuity, Pattern Electroretinogram (PERG), visual evoked potential (VEP) parameters, compared with pre-treatment values. The results persisted after the wash out period, compared to baseline.

**Neuroprotective role of Citicoline in glaucoma – Current Evidence**

Physiopathology of glaucoma and neuroprotection

Glaucoma is a group of optic neuropathies characterized by death of retinal ganglionar cells (RGC), which leads to structural and functional abnormalities of the optic nerve. It is the second cause of blindness in the world with 111.8 million people estimated to be affected in 2040. The most important factor is an elevated intraocular pressure (IOP), but hypotensive therapy alone is not sufficient in some cases to preserve the visual function, and the disease continues to progress despite a well-controlled IOP. It is currently recognized as a chronic neurodegenerative disease, which alters the whole visual pathways running from the eye to the visual cortex. This suggests that the pharmacological approach used in different degenerative brain disorders can also be useful in glaucoma. 

In 2006, Gupta first demonstrated the presence of degenerative changes in lateral geniculate nucleus and visual cortex in glaucoma patients. The eye could be part of the central nervous system and glaucoma could be a neurodegenerative disease. There are also common cell death mechanisms of glaucoma and neurological progressive diseases [**16**].

The death of RGC is the main pathophysiological event in glaucoma and neuroprotection has the aim to prevent, delay, or reduce the cell death by targeting the neurons.

One of the mechanisms involved is the deprivation of neurotrophins. Acute or chronic IOP elevation can cause a blockade of the axonal transport of neurotrophins from the superior colliculus to the optic nerve head. Apoptosis can be the result of neurotrophic factors deprivation, such as brain derived neurotrophic factor (BDNF), which are important for cell survival and growth. Glutamate-mediated toxicity is another mechanism that has been investigated; blocking glutamate cascade could represent a neuroprotective strategy.

## Experimental studies

In recent years, experimental studies have proven the protective role of Citicoline on RGCs.

In 2002, Rejadak et al. [**17**] showed the impact of Citicoline on retinal catecholamine levels. He injected intraperitoneally adult male Albino rabbits and measured the concentration of catecholamines in the retina, showing a higher level of dopamine in the animal treated with Citicoline, a slightly higher adrenaline concentration, while noradrenaline was unmodified.

Citicoline could also have an anti-apoptotic effect in the mitochondria-dependent cell death mechanism and could help the axon regeneration. Shuettauf et al. [**18**] studied the anti-apoptotic effect of Citicoline in adult rats by treating them with lithium, Citicoline or a mixture of lithium and Citicoline by intraperitoneal injections. The results indicated a higher density or RGC connected with the superior colliculus in those treated with Citicoline compared with the others.

**Fig. 3:**
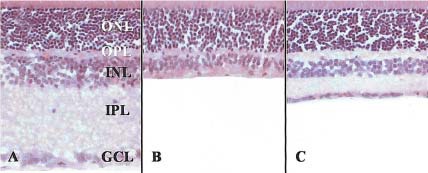
Transverse sections in rat retina at 7 days after KA injection using H-E staining. In control retina, five, well organized retinal layers are seen (A). In the KA-injected group, the thickness of retina is markedly reduced due to loss of internal nuclear (INL) and internal plexiform layers (IPL) (B). Conserved retinal layers in KA-injected group treated with Citicoline (C)

Some authors demonstrated the neuroprotective role of Citicoline in glutamate-mediated cell death by using a model of Kainic acid (KA)-induced retinal damage in rats, which is an analogue of glutamate. They injected KA in the vitreous space and Citicoline intraperitoneally in some of the rats. Compared to the control group that received only KA, in which a reduction of retinal thickness was noticed gradually, the group treated with Citicoline showed an attenuated reduction (**Fig. 3**) [**19**].

The neuroprotective effect of Citicoline on retinal nerve fibers in hyperglycemia conditions has also been proven by using Citicoline eye drops in a mouse model of diabetes [**20**].

## Clinical studies

Many studies have investigated the neurotrophic effect of Citicoline in glaucoma. The effects of Citicoline treatment in glaucoma were analyzed by perimetry using Humphrey Field Analyzer and electrophysiological methods. The last can describe the structures that contribute to the visual function. The function or retinal ganglionar cells are analyzed by pattern electroretinogram (ERGp). Visual evoked potentials (VEP) describe the whole visual pathways.

In 1999, Parisi et al. [**21**] investigated the effect of Citicoline by using electrofunctional tests (VEP and ERGp) in order to assess the retinal and cortical response in patients with glaucoma. The intramuscular dose of Citicoline or placebo was added to their hypotensive treatment, followed by a washout period. The Citicoline-treated group showed an improvement of VEP and ERGp parameters when compared to placebo that was treatment-dependent.

In 2005, Parisi et al. [**22**] evaluated the long-term treatment effect of Citicoline treatment in a 8 years study adding Citicoline to the hypotensive therapy followed by a washout period and repeated the protocol for the whole period. The results obtained at the end of each period were compared to baseline. Citicoline improved the VEP and ERGp parameters compared to pre-treatment conditions and to placebo patients. An increase in the visual field mean deviation was also observed at the end of the follow up, and this was linked to the electrofunctional results. This improvement could be determined by dopamine increase in the central nervous system. 

All the above studies refer to Citicoline administered by intramuscular injection. In 2003, Rejadak et al. [**23**] made the first clinical study by using Citicoline tablets containing 500 mg of active ingredient, given twice a day. VEP measurement showed an improvement of conduction along the visual pathways.

**Fig. 4:**
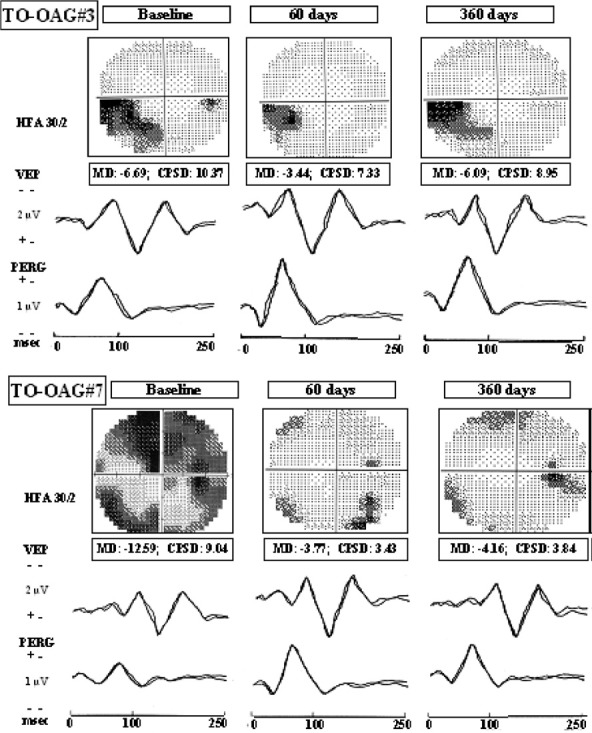
Electrophysiological examination at baseline, after 60 days of treatment with Citicoline and after the last washout period. Improvement of visual field and electrophysiological parameters after treatment followed by regression or stabilization after washout [**24**]

In 2008, Parisi et al. [**24**] studied the effect of oral suspension of Citicoline versus the intramuscular administration over visual function in patients with moderate visual field defects. They noticed an improvement of VEP and ERGp parameters after both treatments, with no difference between the two administration routes. After washout, a partial regression was noticed (**Fig. 4**).

Citicoline can also be administered as eye drops in order to increase the patient compliance and adherence. In an experimental study, Citicoline has been detected in the vitreous when administered topically [**25**]. In this study, five mice were treated with Citicoline eye drops 1% and 2%, two drops per day. The molecule was detected in vitreous at the end of the treatment. When 2% Citicoline was administered, a systemic absorption was also noted. This study had a clinical part too. The authors added Citicoline eye drops to the hypotensive therapy of glaucoma patients for 2 months followed by one month of washout. After the first 2 months of treatment, an improvement in RGC function was noted on electrofunctional tests, but regressed after 30 days of washout.

The positive effects of Citicoline on the retinal function and neural conduction in glaucoma patients was also reported by Parisi et al. [**26**]. They treated a group of patients with beta-blocker monotherapy and Citicoline eye drops 3 times per day for 4 months followed by a 2 months washout period, and the other group with beta-blocker monotherapy for the whole period. The electrofunctional measurements were done at the baseline, at four and six months. An improvement was noticed after the 4 months of Citicoline treatment compared to baseline, and the increase of VEP P100 was correlated with the increase of ERGp parameters. The results went back to the baseline levels after washout period. The group treated with beta-blockers only maintained the same electrophysiological level during the whole study.

## Conclusions and Future Perspectives

There are 3 steps of neuroprotection in glaucoma: to protect unaffected axons and RGC, to save minimally damaged axons and RGC and to regenerate them. Citicoline may play a role in the second step, acting between the dysfunction and the apoptosis of RGC. In animal models, the steps of RGC death were demonstrated, which occur later in the disease course: a decrease in axonal transport, a split of axons and the death of RGC later [**27**]. The time between neuronal dysfunction and death could be used as a good moment to introduce therapies in order to increase the retinal function (neuroenhancement), and Citicoline may play a role in this process.

The scientific literature regarding the Citicoline role in glaucoma is growing and the studies differ in terms of patients’ characteristics, outcomes measures, schedule of treatment (dosage, administration route, length). The conclusion of all the studies made so far is that Citicoline is a safe molecule with positive effects on the visual function.

In the future, more clinical trials are needed with a larger population involved and more investigations need to be done (optical coherence tomography measurement of retinal nerve fiber layer, ganglion cell complex thickness) in order to obtain a dose-response relation and to sustain the clinical effect of Citicoline demonstrated until now.

**Disclosures**

None
